# Discovery of a drug to treat airway mucus hypersecretion

**DOI:** 10.1002/ctm2.972

**Published:** 2022-07-31

**Authors:** Burton F. Dickey, Ying Lai, Manfred Frick, Axel T. Brunger

**Affiliations:** ^1^ Department of Pulmonary Medicine University of Texas MD Anderson Cancer Center Houston Texas USA; ^2^ National Clinical Research Center for Geriatrics, West China Hospital, State Key Laboratory of Biotherapy and Collaborative Innovation Center of Biotherapy Sichuan University Chengdu China; ^3^ Institute of General Physiology Ulm University Ulm Germany; ^4^ Department of Molecular and Cellular Physiology Stanford University Stanford California USA; ^5^ Howard Hughes Medical Institute Stanford University Stanford California USA

1

Pathologists have recognized the central role of airway mucus occlusion in the pathophysiology of asthma for more than a century,^1^, ^2^ and in cystic fibrosis for nearly a century.^3^ In chronic obstructive pulmonary disease (COPD), understanding of the role of mucus centred for many years around its expectoration from central airways as the defining feature of the chronic bronchitic endotype, though expectoration correlates only weakly with airflow limitation.^4^ However, more recent examination of peripheral lung tissues from surgical specimens revealed that the mucus occlusion of small airways correlates strongly with airflow limitation.^5^ Despite this recognition by pathologists, clinicians have largely ignored the role of mucus in these obstructive lung diseases because airway occlusion could not be evaluated non‐invasively and available treatments have been limited. Recent reports that chest CT scans could demonstrate occluded airways in asthma and COPD, and that such occlusion correlates with poor clinical outcomes, has raised interest in the muco‐obstructive endotype of airway diseases.^6^, ^7^ In addition, multiple therapies for mucus occlusion have recently become available or are in development, further raising the value of diagnosis and treatment.^2^, ^8^, ^9^

**FIGURE 1:**
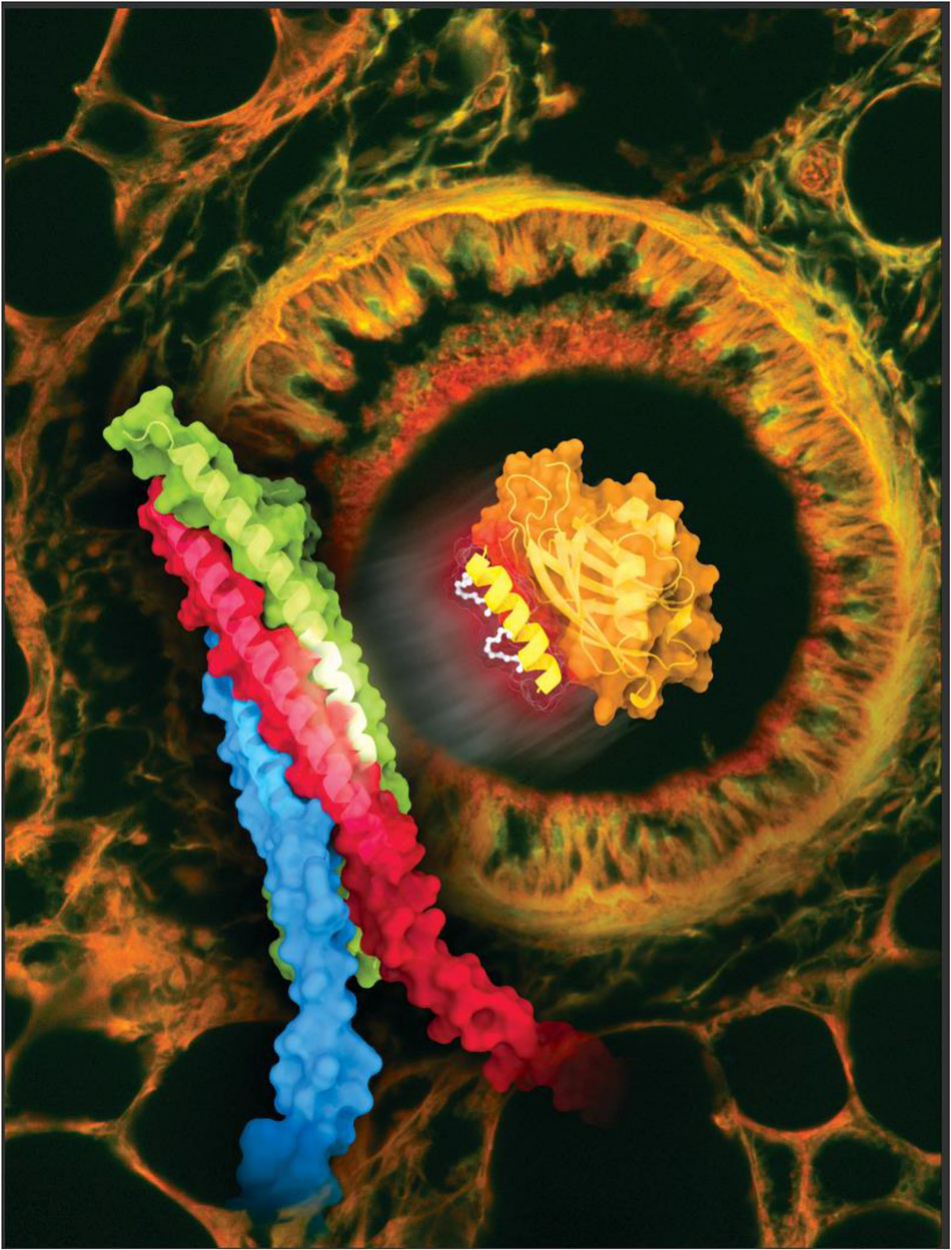
Inhibition of stimulated mucin secretion by a stapled peptide. Foreground: the stapled peptide SP9 (bright yellow, with white staples) disrupts the interaction of the calcium sensor synaptotagmin‐2 (Syt2) (orange) with the SNARE complex (green, red and blue) responsible for exocytosis. The white segment of one of the green helices is cognate to SP9. Background: a transverse section of a mouse bronchial airway showing intracellular and extracellular mucin (red). Administration of the stapled peptide keeps the airway mostly free of mucus occlusion. *Source*: Illustration by Eric D. Smith

Mucus is a gel‐like substance comprising mucin glycoproteins (.3%), salts (2%) and water (97%), along with various entrapped globular proteins (.7%) more conventional to write 0.3% and 0.7%. Importantly, the intracellular packaging of mucins in anhydrous form demands that they absorb 300‐fold their mass of water after secretion to yield mucus of optimal viscoelasticity. Normally, mucus is moved proximally in the airways and eventually out of the lungs by beating cilia, removing inhaled particles and pathogens in an essential defence mechanism.^2^ However, high levels of mucin production from inflammatory stimulation (termed ‘mucous metaplasia’), followed by rapid release (together, termed ‘mucus hypersecretion’), can plug airways due to mucus volume expansion. In addition, if available lumenal liquid is insufficient, concentrated mucus of excessive viscoelasticity and adhesivity can cause mucus stasis.^4^ In principle, there are several therapeutic avenues to address mucus occlusion, including blocking upstream inflammatory mediators that promote mucin production, suppressing mucin synthesis by manipulating Notch or MAPK13 signalling or by RNA interference, promoting autophagy of mucin before it is secreted, altering mucus viscoelasticity by hydration or the use of mucolytics, and inhibiting rapid mucin secretion.^2^, ^9^, ^10^ Of these, the last is particularly tricky because of the need to preserve steady homeostatic (baseline) release of mucins for ciliary clearance, while preventing the explosive (stimulated) release of abundant stored mucins. Recently, four longstanding lines of research came together to allow the synthesis of a compound that threads the therapeutic needle between baseline and stimulated mucin secretion (see Figure 1).^11^

The first line of enabling research was the molecular dissection of the mechanism of exocytic secretion. This began in the 1970s with a genetic screen in yeast that identified many proteins mediating vesicle traffic on the secretory pathway, including the core SNARE machinery and key interactive proteins.^12^ However, yeast have only a constitutive secretory pathway, so the identification of regulatory components that enable stimulated secretion required studies in metazoans. Neurons have been the most intensively studied regulated secretory cells, and their calcium sensor was identified as synaptotagmin‐1 (Syt1).^12^ To elucidate the exocytic mechanism in airway secretory cells, we and others sought orthologs of the neural mechanism.^13^ The identification of Syt2 as the calcium sensor in stimulated but not baseline mucin secretion made it a candidate therapeutic target.^14^

The second line of enabling research was the elucidation of high resolution structures of interactions of Syt1 with the core membrane fusion machinery consisting of neuronal SNAREs.^15^, ^16^ All these structures revealed a structurally and functionally conserved interface (called the ‘primary interface’) that mostly involves parts of the SNAP‐25 and syntaxin SNARE proteins. Based on the structure of this interface, certain fragments of SNAP‐25 were predicted to be capable of competing with the SNARE complex for interaction with Syt1.

Complementing advances in structural insight into the mechanism of secretion were functional insights obtained from biochemical reconstitution. This third line of enabling research had begun with fractionated Golgi membranes and cytosol in the 1970s in parallel with the genetic dissection of the secretory pathway in yeast.^12^ Those two approaches identified many of the same proteins and provided complementary insights into molecular functions. In recent years, biochemical reconstitution was refined by using purified proteins and synthetic liposomes together with advanced single particle optical microscopy.^15^ Moreover, the cytoplasmic regulatory proteins Munc18 and Munc13 were included along with the SNARE proteins and Syt1 that were reconstituted into synthetic liposomes, bringing the system to a more physiological state.^16^ These advanced reconstitution assays allowed the confirmation of the functional importance of critical amino acids involved in calcium‐triggered membrane fusion suggested by the structural work.

The fourth line of enabling research was advances in protein chemistry allowing the stabilization of the secondary structure of peptides and their delivery into the interior of cells. Peptide fragments in isolation often lack the secondary structure of the corresponding region of the parent protein. A powerful technology to constrain peptide conformation is hydrocarbon stapling, by which a subset of amino acids are covalently linked by olefin (alkene) bridges in addition to their primary linkage via the peptide backbone.^17^ Work in this area began in the 1960s in the petrochemical industry with the recognition that carbon–carbon double bonds could be redistributed within alkene fragments by metal catalysts (‘olefin metathesis’). That work was extended to medicinal chemistry in the 1990s by incorporating non‐natural amino acids containing olefin side chains into therapeutic peptides, then linking them with efficient metal catalysts, resulting in an effective means to stabilize α‐helical structure.^17^ Besides constraining conformation, hydrocarbon stapling can make peptides resistant to proteolysis and somewhat membrane permeant. Despite the latter property of hydrocarbon staples, the efficient delivery of peptides into the cytoplasm generally requires additional measures. In 1988, two groups reported that the transactivator of transcription (TAT) protein of HIV‐1 was taken up by cells, and in 1991, the homeodomain of Antennapedia was also shown to enter cells.^18^ Further analysis of the Antennapedia homeodomain revealed that a short 16‐amino acid peptide, termed penetratin, was responsible for translocation, and further analysis of TAT similarly showed that the transduction motif could be reduced to 9 amino acids. Since then, a plethora of natural and artificial cell‐penetrating peptides have been discovered that can be conjugated to therapeutic peptides to transduce them into cells, and several are in clinical trials.^18^

These four lines of investigation came together in collaborative work that has been recently published.^11^, ^19^ First, Syt2 was validated as a therapeutic target to treat mucus hypersecretion by conditionally deleting it in airway epithelial cells of mice. Deletant mice showed marked reductions in stimulated mucin secretion and lumenal mucus occlusion in a model of allergic asthma, without an impairment of homeostatic baseline mucin secretion.^11^ Next, a series of stapled peptides based on the region of SNAP‐25 known to interact with Syt1 were screened for their ability to inhibit a fusion of reconstituted synaptic vesicles with reconstituted neuronal plasma membranes. Four of eleven peptides with the highest degree of α‐helicity inhibited calcium‐triggered fusion, and one of the most efficacious of these (termed SP9) was also tested in a reconstituted airway secretory system where it again specifically inhibited calcium‐triggered secretion.^19^ Besides stabilizing peptide structure, the staples also reduced undesirable side effects by preventing peptide interactions with the ternary SNARE complex required for baseline mucin secretion and constitutive non‐mucin secretion. Next, SP9 was conjugated to cell penetrating peptides and added to cultured human airway epithelial cells. SP9 conjugated to TAT or penetratin (PEN) and the fluorophore Cy3 efficiently entered the cultured cells, where it inhibited stimulated but not baseline mucin secretion.^11^ Last, SP9 conjugated to these cell penetrating peptides and Cy3 were aerosolized into the airways of mice. TAT‐SP9‐Cy3 artefactually induced mucin secretion, possibly by perturbing membranes to allow cytoplasmic calcium entry, but PEN‐SP9‐Cy3 did not induce this artefact. Mice with PEN‐SP9‐Cy3 aerosolized into their airways before any exposure to a secretagogue were protected from stimulated mucin secretion and lumenal mucus occlusion to a degree similar to Syt2 airway deletant mice.^11^ Together, these results identified PEN‐SP9 as a lead compound for the development of an inhaled therapeutic for the prevention of mucus hypersecretion.

Going forward, it is unlikely that a conventional small molecule will achieve a similar effect because there are no pockets in the primary interface to bind. Rather, multiple hydrophobic and ionic interactions confer binding between SP9 and Syt1/Syt2. Multiple natural and non‐natural amino acid substitutions of SP9 congeners, as well as other approaches besides hydrocarbon staples to stabilize the α‐helical structure, can be considered in optimizing peptide affinity and stability. Peptide mimetic structures can also be considered, as well as alternative means of achieving cell penetration.

Our identification of SP9 was based on extensive structural and functional data on the interaction between Syt1 and the ternary SNARE complex,^15^, ^16^ and the high affinity binding of synthetic proteins to target proteins based only on the target structure has been achieved recently,^20^ together suggesting the potential of peptide/protein drugs to target previously ‘undruggable’ proteins. Besides its potential therapeutic value, our work is of interest because it builds on fundamental research into the molecular mechanism of vesicle traffic performed over the past five decades, as well as advances in protein chemistry made over a similar time frame. As such, it validates the significance of basic research to improve human health.
